# Migrastatics—Anti-metastatic and Anti-invasion Drugs: Promises and Challenges

**DOI:** 10.1016/j.trecan.2017.04.008

**Published:** 2017-06

**Authors:** Aneta Gandalovičová, Daniel Rosel, Michael Fernandes, Pavel Veselý, Petr Heneberg, Vladimír Čermák, Luboš Petruželka, Sunil Kumar, Victoria Sanz-Moreno, Jan Brábek

**Affiliations:** 1 Department of Cell Biology, Charles University, Viničná 7, Prague, Czech Republic; 2 Biotechnology and Biomedicine Centre of the Academy of Sciences and Charles University (BIOCEV), Průmyslová 595, 25242, Vestec u Prahy, Czech Republic; 3 Medbase, Chapel Hill, NC, USA; 4 Central European Institute of Technology, Brno University of Technology, Brno, Czech Republic; 5 Charles University, Department of Internal Medicine, Third Faculty of Medicine, Prague, Czech Republic; 6 Department of Oncology, First Faculty of Medicine, Charles University and General University Hospital, Prague, Czech Republic; 7 Ayurveda Molecular Modeling, Hyderabad, Telangana, India; 8 Tumor Plasticity Laboratory, Randall Division of Cell and Molecular Biophysics, Guy’s Campus, King’s College London, London, UK

**Keywords:** solid cancer, metastasis, invasion, treatment, contractility, migrastatics

## Abstract

In solid cancers, invasion and metastasis account for more than 90% of mortality. However, in the current armory of anticancer therapies, a specific category of anti-invasion and antimetastatic drugs is missing. Here, we coin the term ‘migrastatics’ for drugs interfering with all modes of cancer cell invasion and metastasis, to distinguish this class from conventional cytostatic drugs, which are mainly directed against cell proliferation. We define actin polymerization and contractility as target mechanisms for migrastatics, and review candidate migrastatic drugs. Critical assessment of these antimetastatic agents is warranted, because they may define new options for the treatment of solid cancers.

## Trends

Local invasion and metastasis, rather than clonal proliferation, are the dominant features of solid cancer. However, a specific category of anti-invasion and antimetastatic drugs is missing for treatment of solid cancer

We propose the term ‘migrastatics’ for drugs interfering with all modes of cancer cell invasiveness and, consequently, with their ability to metastasize (e.g., inhibiting not only local invasion, but also extravasation and metastatic colonization).

In solid cancer, drug resistance is the main cause of treatment failure, and is attributed to mutations of the target. Since targeting the cause, although academically desirable, may be futile, a pragmatic and near-term option is to move downstream, to common denominators of cell migration and/or invasion, such as actin polymerization and actomyosin-mediated contractility.

## Migrastatics As Antimetastatic Drugs

Cancer is characterized by abnormal cellular proliferation and the potential to spread to other parts of the body. Hematologic malignancies involve the blood, bone marrow, and lymphatic system, and a predominant feature is uncontrolled clonal proliferation 1, 2. For this reason, cytotoxic drugs have proven to be an effective treatment (reviewed in [3]). By contrast, solid cancer is accompanied by local invasion and metastasis [4]. Treatment of solid cancer should be complemented with drugs that inhibit the ability of cancer cells to invade through the extracellular matrix (ECM) and establish secondary tumors. Since mechanisms determining clonal proliferation, cell migration, and invasion are distinct, it is evident that drug discovery efforts should be dichotomized into antiproliferative strategies and those directed towards mechanisms related to motility, migration and/or invasion, and metastasis. This is important and relevant to translational therapies in solid cancer. Candidate drugs for solid tumors are still evaluated predominantly by their ability to induce tumor shrinkage. Progression in solid cancer is conventionally defined as an increase in tumor size, and, in a superficial sense, the equating of therapeutic efficacy with tumor shrinkage is understandable. However, tumor shrinkage is rarely absolute or sustained, and is not predictive of an antimetastatic effect. Moreover, a focus on dimension detracts from attention to local invasion and metastasis, which account for more than 90% of mortality [5].

The ability to invade and metastasize is a cancer hallmark, as defined by Weinberg and Hanahan [6]. According to Lazebnik [7], the gain of an invasive phenotype is the most important cancer feature and the one that distinguishes malignant from benign tumors. Most morbidity and mortality in solid cancer stem from metastases. Strikingly, this is not reflected in funding and efforts towards antimetastatic research (reviewed in [5]). To date, medicinal chemists continue to focus on antiproliferative agents because tumor shrinkage is a regulatory requirement for approval. However, this approach underestimates the effect on cancer invasion and, as a result, patients and oncologists bemoan the lack of antimetastatic drugs [4].

Here, we introduce the term ‘migrastatics’ (from Latin ‘*migrare*’ and Greek ‘*statikos*’) for drugs interfering with all modes of the invasion of cancer cells and, consequently, with their ability to metastasize. The term is used to emphasize a focus on the inhibition of local invasion and metastasis, and to define and distinguish this class from conventional cytostatic drugs that are mainly directed against cell proliferation. Here, we review mechanisms related to early steps in the process leading to cancer metastasis, namely motility, directed migration, and invasion of the transformed cancer cell. Furthermore, we provide examples of relevant natural products and a rationale for their role as migrastatic candidates. Recently identified synthetic migrastatics candidates are also discussed. To finish, we discuss toxicity and clinical implications of migrastatics.

## Requirements for the Implementation of Migrastatics

For the successful establishment of migrastatics, two main requirements need to be considered: (i) fine-tuning regulations for the approval of anticancer drugs. An emphasis on antimetastatic effects (related mainly to the inhibition of cancer cell motility and invasiveness) will allow clinical evaluation of candidate drugs even in the absence of tumor shrinkage (a point addressed elsewhere 4, 8). A precedent has already been set with checkpoint inhibitors [9]; and (ii) large-scale testing of compound libraries as well as a search for new compounds to select drugs that display low toxicity and interfere with all modes of cancer cell motility in 3D systems and animal models.

Although we propose here migrastatics as an independent class of drugs, it should be noted that there is ‘nothing new under the sun’. In broad evolutionary terms, antimigratory and/or anti-invasive mechanisms are likely to have evolved as defensive measures, and migrastatics may be produced by several species of animals, plants, and microorganisms. Understandably, toxicity is a key concern with botanical product-derived candidates, and bioassay-guided fractionation of promising natural products has been helpful to identify promising pharmacophores [10]. Recent medicinal chemistry efforts based on cell biology have now defined attractive candidates for drug development [11].

## Cancer Cell Invasion: A Target in Antimetastatic Intervention

**Figure 1:**
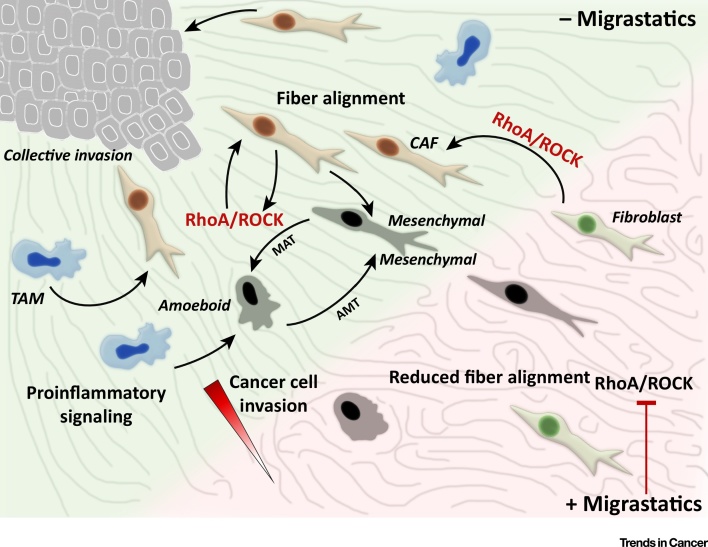
The Plasticity of Cancer Cell Invasion. Cancer cells can invade either collectively or as individual cells when utilizing the amoeboid or mesenchymal invasion mode. Cells invading in one mode can undergo the mesenchymal-amoeboid, or amoeboid-mesenchymal mode (MAT and AMT, respectively) in response to current conditions and signaling within the extracellular matrix (ECM). The plasticity of invasion is further regulated by interactions with noncancer cells that contribute to signaling circuits. Tumor-associated macrophages (TAMs) produce proinvasive cytokines that affect invasion directly and sustain the cancer-associated phenotype of proximal fibroblasts. These cancer-associated fibroblasts (CAFs) realign fibers of the ECM, which facilitates cancer cell invasion. The Rho/Rho-kinase (ROCK) pathway is crucial for many of these interactions and, thus, its inhibition downregulates cancer cell invasion (for more details, see the main text).

During dissemination from a primary tumor, cancer cells invade the ECM most commonly in clusters or as sheets [12], which is referred to as ‘collective migration’. This requires proteolytic degradation at the leading edge of the invasive front and cell contractility in the following cells [13]. Alternatively, single cancer cells can detach and invade using protease-dependent mesenchymal migration or protease-independent amoeboid migration, or a combination of both (Figure 1). Furthermore, many cancer cells can actively switch between these invasion modes in response to changes in the surrounding environment and/or to escape therapy (reviewed in 14, 15, 16).

For example, the use of matrix metalloprotease inhibitors can arrest mesenchymal migration, but does not halt invasion in general, because cells can undergo the mesenchymal-amoeboid transition (MAT) and switch to protease-independent invasion [17]. Furthermore, MAT was observed after enhancing cell contractility or in loose cell ECM 18, 19. The opposite process, the amoeboid-mesenchymal transition (AMT), can be induced by upregulating Rac activity, which decreases contractility [20].

The plasticity of cancer cell invasion is further promoted by interactions within the tumor stroma, where noncancer cells contribute to signaling circuits regulating invasion. For example, tumor-associated macrophages (TAMs) produce proinvasive cytokines that not only affect invasion directly, but also sustain the cancer-associated phenotype of proximal fibroblasts (reviewed in [21]), which realign fibers of the ECM to facilitate cancer cell invasion (Figure 1).

**Figure 2:**
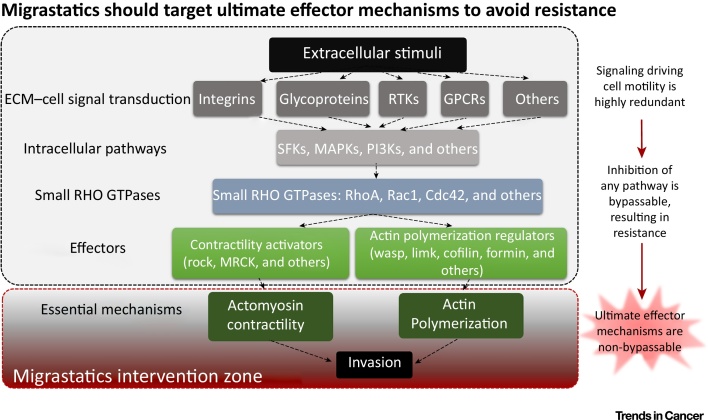
Key Figure: Target Mechanisms of Migrastatics.

An obvious follow-up question is which molecular mechanisms should be targeted by migrastatics? Ideally, it should be those mechanisms that are common and essential for the motility of all migrating cancer cells derived from solid tumors (Figure 2, Key Figure).

Signaling pathways regulating cell migration are highly redundant and inhibition of a single pathway leads almost inevitably to resistance (reviewed in [22]). In fact, resistance itself may explain failures in targeting key, genetically stable mechanisms, since many intracellular signaling processes are redundant. Thus, while precise targeting of suspect pathways is possible, it is unlikely to be successful (Figure 2).

Accordingly, we propose that migrastatics should target the ultimate downstream effector mechanisms of cell migration, such as actin polymerization and contractility, which are difficult to bypass. It is unlikely that cancer cells will be able to substitute actin polymerization or develop an alternative contractile apparatus. Actin polymerization and contractility satisfy the requirement for ideal migrastatics targets because these processes are required by all invasion and/or 3D migration mechanisms irrespective of their protease dependence.

In general, the migrating cancer cell is characterized by cellular shape rearrangements involving the formation of actin-based protrusions and new adhesions to surfaces, as well as cellular contractility, which is required for rear retraction and cell body translocation 23, 24. The actin cytoskeleton has a crucial role and undergoes constant reassembly during all these processes [25]. Actin also participates in the formation of specialized invasive structures, such as invadosomes, which are adhesive structures with proteolytic activity formed by mesenchymally migrating cells at the cell–ECM interface [26].

**Figure 3:**
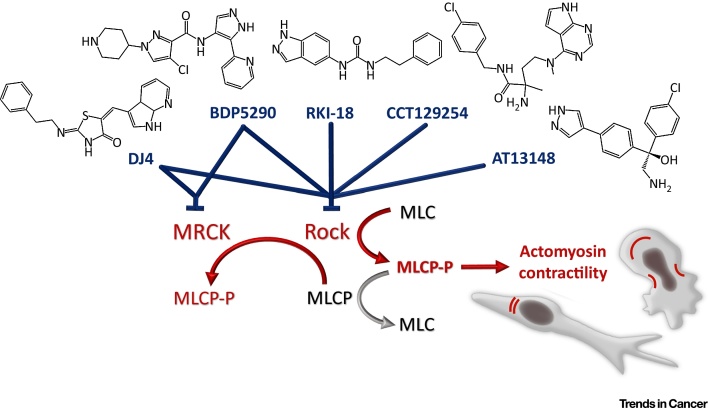
Regulators of Actomyosin Contractility Are Targets for Migrastatics. Rho-kinase (ROCK) mediates the phosphorylation of myosin light chain (MLC) to directly enhance contractility. In addition, ROCK and myotonic dystrophy kinase-related CDC42-binding kinase (MRCK) phosphorylate and, thus, inhibit MLC phosphatase (MLCP), which counteracts MLC phosphorylation. Thus, drugs targeting ROCK or MRCK are candidates for efficient migrastatics because they act to inhibit actomyosin contractility, which is necessary for of all cell invasion modes. Candidate drugs are depicted in blue, whereas enhancers of actomyosin contractility are in red.

In cooperation with myosin motors, actin is the key resource for cellular contraction. Together, they form a meshwork that assembles into various structures, such as the contractile ring in the case of cytokinesis, sarcomeres in muscle cells, stress fibers and/or blebs in migrating cells, or bundles found at the cell cortex 27, 28, 29. The main event regulating actomyosin contractility is the Rho-driven activation of Rho-kinase (ROCK), which directly phosphorylates myosin light chain (MLC) [30]. Furthermore, ROCK as well as myotonic dystrophy kinase-related CDC42-binding kinase (MRCK), phosphorylate myosin light chain phosphatase (MLCP), which leads to its inactivation 31, 32. Phosphorylation of both MLC and MLCP results in increased levels of phosphorylated MLC, which promotes its ATPase activity, resulting in actomyosin contractility (Figure 3).

The importance of the actin cytoskeleton during metastasis is reflected at the level of actin-binding proteins because many of these are deregulated in metastatic cells 33, 34. So far, the use of compounds targeting cytoskeletal dynamics has been neglected due to the abundance and importance of cytoskeletal components and possible adverse effects. However, the successful clinical use of microtubule-binding agents as anticancer drugs weakens this argument [35]. While the evaluation of some microtubule-binding agents has been discontinued because of significant toxicity, others have become drugs with crucial importance for cancer treatment, particularly vinca domain-binding agents (vincristine, vinblastine, vinorelbine, vindesine, and vinflunine) and taxol domain-binding agents (paclitaxel, docetaxel, and cabazitaxel) [35]. Moreover, natural products targeting the cytoskeleton as well as synthetic drugs deemed too potent to elicit therapeutic benefits can now be conjugated to an appropriate protein delivery system, thereby delivering highly cytotoxic and specific treatments to neoplastic tissue.

## Candidate Migrastatic Drugs

### Drugs Targeting Actin Polymerization and Function

**Figure 4:**
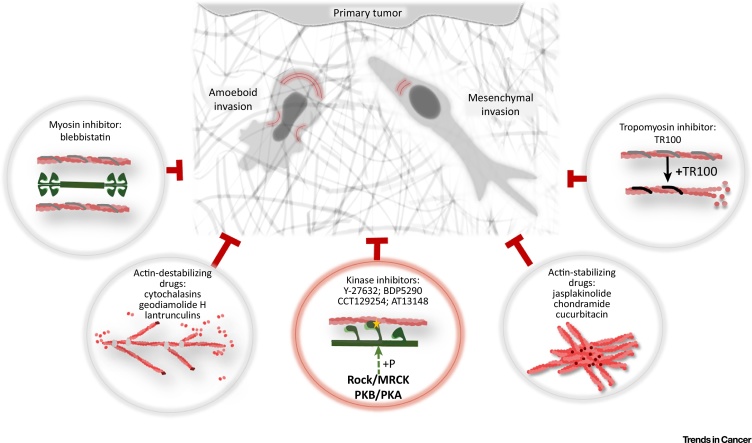
Potential Candidates for Migrastatics. Drugs targeting the actin cytoskeleton are suitable candidates for the inhibition of cell invasion because they impair both amoeboid and mesenchymal invasion. Chosen groups of migrastatic agents are depicted. Drugs interfering with actin dynamics include actin cytoskeleton-destabilizing drugs (cytochalasins, latrunculins, and geodiamolide H) and actin filament-stabilizing drugs (jasplakinolide, chondramide, and cucurbitacin). TR100, a tropomyosin inhibitor, disrupts the actin cytoskeleton by affecting its stability. Other drugs target actomyosin contractility, such as blebbistatin (an inhibitor of non-muscle myosin II) or inhibitors (e.g., Y-27632, BDP5290, CCT129254, or AT13148) that target kinases involved in the regulation of actomyosin contractility. The group of kinase inhibitors is emphasized because they have shown the potential to inhibit cell invasion in *in vivo* experiments. For more detail on certain drugs, refer to the main text.

Whereas the actin cytoskeleton is a crucial component involved in cancer cell migration, agents targeting actin dynamics have been relatively poorly investigated (reviewed in [36]; see also 37, 38). Consequently, *in vitro* pharmacological tools are needed to selectively identify this type of agent [39]. These drugs can be categorized as compounds that destabilize the actin cytoskeleton (e.g., cytochalasins, geodiamolides, and latrunculins) and compounds that stabilize actin filaments, initiate deregulated polymerization, monomer depletion, and formation of large actin aggregates (e.g., jasplakinolide, chondramide, and cucurbitacin E) (Figure 4). Migrastatic drug candidates targeting actin polymerization and function, including evidence that these drugs effectively inhibit cancer cell invasion and/or metastasis, are discussed further below and in Table 1.

**Table 1:** Selected Migrastatic Candidates

Structure	Target	Activity	Models	Refs
	G-actin; interaction with thymosin β4	>95% inhibition of invasiveness at 100 ng/mL; ↓ invasiveness	AMDC-S and AMDC-AS cell lines	[53]
Latrunculin A			G3S1 cells	[54]
	Actin	↓ Invasiveness (<50% at 30 nM); ↓ phosphorylation of MLC2; ↓ contractility	MDA-MB-231 cells	[62]
Chondramide				
	Tropomyosin	EC_50_ = 1.9 uM	SK-MEL-28 cell line	[68]
		EC_50_ = 4.1 uM	Melanoma cell lines	[68]
		EC_50_ = 2.8 uM	Pediatric tumor cell lines	[68]
TR100		↓ Invasiveness	Melanoma cell lines	[68]
	ROCK1	IC_50_ = 397 nM	MDA-MB-231 cells	[119]
	ROCK2	IC_50_ = 349 nM		
RKI-18		↓ Invasiveness		
	ROCK1	IC_50_ = 230 nm	MDA-MB-231 cells	[120]
		EC_50_ = 501 nm at 0–3 μM		
	ROCK2	IC_50_ = 123 nm		
		EC_50_ = 447 nm at 0–3 μM		
	MRCKα	IC_50_ = 10 nm		
		Ki = 10 nm		
	MRCKβ	Ki = 4 nm		
		EC_50_ = 166 nm at 0–3 μM		
BDP5290		↓ invasiveness; ↓ phosphorylation of MLC		
	ROCK1	IC_50_ = 5 nM	NSCLC cell lines	[121]
	ROCK2	IC_50_ = 50 nM	H522, MDA-MB-231, and PANC-1 cell lines	[121]
	MRCKα	IC_50_ = 10 nM		
	MRCKβ	IC_50_ = 100 nM		
DJ4		Blocked recombinant MYPT1 and MLC phosphorylation at 5 μM; inhibited migration and invasiveness		
	ROCK I	IC_50_ = 214 nM	Melanoma cell lines, mouse	[112]
	ROCK II	IC_50_ = 141 nM		
	AKT2	IC_50_ = 2.2 nM		
		↓ invasiveness; ↓ metastasis; ↓ phosphorylation of MLC2 and AKT		
CCT129254	AGC kinases	>70% inhibition at 1 μM		
	ROCK I	IC_50_ = 6 nM	Melanoma cell lines, mouse	[112]
	ROCK II	IC_50_ = 4 nM		
	AKT1	IC_50_ = 38 nM		
	AKT2	IC_50_ = 402 nM		
	AKT3	IC_50_ = 50 nM		
		↓ Invasiveness; ↓ phosphorylation of MLC2 and AKT		
AT13148	AGC kinases	>70% inhibition at 1 μM		

### Drugs Destabilizing Actin Cytoskeleton

Cytochalasins are drugs interfering with actin polymerization characterized by a highly substituted perhydro-isoindolone structure that is attached to a macrocyclic ring. More than 60 different cytochalasins from several species of fungi have been classified into various subgroups based on the size of the macrocyclic ring and the substituent of the perhydroisoindolyl-1-one residue at the C-3 position [40]. Despite this diversity, only cytochalasins B and D have been extensively studied for their chemotherapeutic potential. Cytochalasin D was shown to not only inhibit invasion of AGS gastric cells, particularly after induction with LPA [41], and MDA-MB-231 breast carcinoma cells [42], but also to promote pulmonary metastasis of B16 melanoma through the expression of tissue factor [43]. Many studies that have examined the anticancer activity of cytochalasins concentrated their efforts on cytochalasin B because it appears to be a safer and less toxic alternative to the more potent cytochalasin D [44]. The antimetastatic effects of Cytochalasin B have been well known since the late 1970s [45]. It was shown to inhibit the metastasis of mouse B16-F10 mouse melanoma cells [46] and Madison 109 mouse lung carcinoma cells [47]. In the latter, an immunosuppressive effect of cytochalasin B was observed, although the same group later showed that this immunosuppression could be completely abolished through the introduction of human recombinant interleukin-2 [48].

Geodiamolides are actin-targeting drugs that disrupt actin filaments and are derived from marine sponges. These compounds are cyclodepsipeptides and have the ability to potently stabilize actin fibers in a manner comparable with phalloidin; however, in contrast to phalloidin, they are freely cell permeable, rendering them exciting targets for drug development (reviewed in [49]). Geodiamolide H was shown to inhibit invasiveness of human breast cancer Hs578T cells when tested *in vitro* at concentrations of 60–120 nM [50].

Latrunculins are microfilament-directed agents, also derived from marine sponges, that inhibit actin polymerization through the sequestration of G-actin monomers [51]. The compound structure is a 14- or 16-membered macrolide base attached to a 2-thiazolidinone moiety [52]. Latrunculin A was found to inhibit the invasion of the tumorigenic AdoMetDC transformants of murine fibroblasts [53], the human breast cancer G3S1 cell line [54] and HeLa-O3 cells [55]. Latrunculin A and its derivatives, latrunculin A-17-O-carbamates, inhibited the invasiveness of human prostate cancer PC3 cells and T47D breast carcinoma cells [56]. Other semisynthetic derivatives of Latrunculin A (acetylated, esterified, and *N*-alkylated) exhibited anti-invasive effects against MDA-MB-231 cells [57]. Latrunculin A also inhibited the peritoneal dissemination of human gastric carcinoma MKN45 and NUGC-4 cells [58], making it a good candidate for a migrastatic drug against carcinoma cells.

### Drugs Stabilizing Actin Cytoskeleton

Another actin-targeting drug derived from marine sponges is jasplakinolide, which promotes actin polymerization and stabilizes actin filaments. Its binding to F-actin is competitive with phalloidin [59]. Jasplakinolide is a cyclodepsipeptide containing a tripeptide moiety linked to a polypeptide chain [59]. It was found to reduce lung metastases of systemic Lewis lung carcinoma [60].

Chondramides are cyclodepsipeptides isolated from the myxobacterium *Chondromycescrocatus crocatus* [61]. Their binding to F-actin is competitive with phalloidin. Chondramides inhibit the invasion of human MDA-MB-231 breast carcinoma and inhibit metastasis of 4T1 breast carcinoma cells to the lung without acute toxicity [62], which supports their role as a migrastatic drug.

Cucurbitacin E, a natural product of plants from the family Cucurbitaceae, inhibits the depolymerization of actin filaments by specifically binding to filamentous actin, forming a covalent bond at residue Cys257 [63]. In animal experiments, intraperitoneal administrations of cucurbitacin E significantly inhibited breast tumor metastasis to the lung without affecting apoptosis or proliferation of inoculated 4T1 and MDA-MB-231 breast cancer cells [64].

### Drugs Targeting Contractility

Actomyosin contractility is required for both cell deformability and rear retraction, key mechanisms in amoeboid and mesenchymal invasion, respectively (reviewed in 14, 65; Figure 3). Accordingly, there is clear evidence for a role of ROCK/MRCK/MLC activation in enhancing tumor cell invasion and metastasis via direct effects on amoeboid or mesenchymal cancer cell invasion [66] and/or via indirect effects on cancer-associated fibroblasts to increase ECM stiffness and facilitate cancer cell movement 65, 67 (Figure 1). As described in detail below, there is increasing evidence that inhibiting contractility chemically decreases cancer cell invasiveness and metastasis.

Contractility targeting drugs can be categorized as inhibitors that target actin (chondramides), tropomyosin (TR100), myosin (blebbistatin), MLC kinase (MLCK) (ML-7 and ML-9), ROCK (e.g., fasudil, Y-27632, H-1152, Wf-536, RKI-1447, and RKI-18), MRCK (e.g., BDP5290), ROCK/MRCK (e.g., DJ4) and ROCK/PKA/PKB (e.g., CCT129254 and AT13148) (Figure 4).

### Tropomyosin Inhibitors

A novel class of anti-tropomyosin compounds has been developed that preferentially disrupt the actin cytoskeleton of tumor cells, thus impairing tumor cell motility. The lead compound, TR100, is effective *in vitro* and *in vivo* in reducing melanoma cell invasive outgrowth and tumor cell growth in neuroblastoma and melanoma models at a low micromolar range. Importantly, in testing for potential adverse effects of the treatment, TR100 was shown to have no adverse impact on cardiac structure and function in a mouse xenograft model [68], making it a good candidate for a migrastatic drug.

### Myosin Inhibitors

Blebbistatin is a 1-phenyl-2-pyrrolidinone derivative capable of inhibiting non-muscle myosin II activity. It was shown to inhibit the invasiveness of pancreatic adenocarcinoma [69], mesenchymally invading BE human colon carcinoma cells and MDA-MB-231 human breast carcinoma cells [32], 501mel melanoma cells [70], 4T1 breast cancer cells [71], MCF7/6 breast cancer cells [72], A337/311RP rat and PR9692 avian sarcoma cells [66], and D54 glioblastoma cells [73]. However, no *in vivo* data are yet available for blebbistatin.

### MLCK Inhibitors

MLCK contributes to cell migration by phosphorylating MLC, mainly at the cell cortex [74]. Inhibition of MLCK by its specific inhibitors, ML-7 and ML-9, reduces the invasiveness of human pancreatic cells [75] and rat prostatic cells [76]. Moreover, ML-7 is able to retard the growth of tumors *in vivo* [77].

### ROCK Inhibitors

ROCK is a member of of the AGC kinase family, along with PKA, PKC, and AKT. It has two isoforms that share significant structural specificity and differ mainly in their tissue distribution [78]. All listed ROCK inhibitors are isoform unspecific and act as type I kinase inhibitors, in that they competitively bind the ATP-binding site during the open (active) conformation. However, they differ in their specificity against other members of the AGC family (for IC_50_s, refer to Table 1).

Fasudil was shown to decrease lung metastasis of HT1080 sarcoma cells [79] and was also found to inhibit the LPA-induced invasiveness of human ovarian cancer cells [80], human lung cancer A549 cells [81], *in vitro* and *in vivo* invasiveness of T98 and U251 human glioblastoma cells [82], invasiveness of 95D human lung adenocarcinoma [83], NCI-H446 human small cell lung cancer cells [84], human high metastatic liver cancer cells HCCLM3 [85], and human oral squamous cell carcinoma SCC-4 cells [86]. Of relevance for potential future clinical applications is the fact that fasudil has been clinically approved for treatment of cerebral vasospasm in Japan since 1995 [87].

Y-27632 was the first published selective ROCK inhibitor [88]. It was shown to decrease the invasive activity of rat hepatoma MM1 cells and their dissemination in the peritoneal cavity [89]; inhibit the metastatic growth of human prostatic cancer PC3 cells in immune-compromised mice [90]; decrease intrahepatic metastasis of primary human hepatoma LI7 cells [91]; decrease the bombesin-stimulated invasiveness of Isreco 1 human colon carcinoma cells [92]; and decrease the invasiveness of human MDA-MB-231 breast carcinoma cells [93], A375m2 and WM266.4 human melanoma cells, LS174T human colon carcinoma cells [19], LPA-induced invasiveness of human hepatoma SMMC-7721 cells [94], human anaplastic thyroid cancer ARO cells [95], shear stress-induced invasiveness of human esophageal cancer OC-1 cells [96] and VMRC-LCD human non-small-cell lung cancer cells [97]. In addition, Y-27632 significantly inhibited intrahepatic metastasis orthotropic implantation of CBO140C12 HCC tumor fragments into mice liver [98], and decreased the invasiveness of B16F1 mouse melanoma cells; UvMel 1.3, UvMel 1.5, and UvMel 270 human uveal melanoma cells [99]; PRL-1-expressing A549 human lung carcinoma cells [100]; AMFR-induced motility of esophageal squamous carcinoma cells [101]; LPA-induced invasiveness of human ovarian cancer CAOV-3 and PA-1 cells [102]; SGC-7901 human gastric carcinoma cells [103]; human colorectal carcinoma SW620 cells [104]; U87MG human glioma cells [105]; human hepatocellular carcinoma cells [106]; metastases of HT29 human colorectal carcinoma cells in an orthotropic mouse model of liver metastasis [107]; Y79 human retinoblastoma cells [108]; and Tca8113 and CAL-27 human tongue squamous cell carcinoma cells [109].

However, it was also shown that Y-27632 increased the invasiveness of human glioma U87 and U251 cells [110] and also enhanced the invasion of human gastric carcinoma OCUM-2MD3 cells [111]. Time-lapse microscopy showed conversion of OCUM-2MD3 cells from a round to a more elongated morphology in the presence of Y-27632, and the expression of membrane-type 1 matrix metalloproteinase (MT1-MMP) was elevated, suggesting that inhibition of the RhoA/ROCK pathway undergoes AMT. Y-27632 is less potent than other more recently developed ROCK inhibitors, such as H1152, AT13148 or GSK269962 [112]. Together, results obtained with this compound could be indicative of only the partial inhibition of ROCK kinase activity. Such partial inhibition may still lead to enough actomyosin contractility to allow migration in some cellular systems. Nevertheless, these studies indicate that the contribution of Rho/ROCK signaling to cancer cell migration may vary depending on the cell line tested and on the surrounding microenvironment [113].

H-1152 is a membrane-permeable inhibitor with high specificity for ROCK over other kinases of the AGC family [114]. It was shown to decrease the invasiveness of human breast carcinoma TMX2-28 [115].

Wf-536 was found to inhibit the invasiveness and metastasis of B16 mouse melanoma cells [116] and LLC mouse Lewis lung carcinoma cells [116]. Notably, while Wf-536 has an IC_50_ for ROCK-II of 200 nM, the IC_50_ of its pyrrolopyridine derivative for ROCK-II is as low as 3.6 nM [117].

RKI-1447 and RKI-18 were both found to inhibit the invasiveness of human breast carcinoma MDA-MB-231 cells 118, 119.

### MRCK Inhibitors

BDP5290 was found to be more effective at reducing MDA-MB-231 human breast cancer cell invasion through Matrigel compared with Y27632. Moreover, the ability of human SCC12 squamous cell carcinoma cells to invade a 3D collagen matrix was strongly inhibited by 2-μM BDP5290 but not by the identical concentration of Y27632, despite equivalent inhibition of MLC phosphorylation [120].

### ROCK/MRCK Inhibitors

Although the first generation of ROCK inhibitors, fasudil or Y-27632, effectively inhibited amoeboid invasiveness, their application occasionally induced AMT and resulted in mesenchymal motility, which requires lower levels of actomyosin contractility. Consequently, these inhibitors failed to block cancer cell invasiveness completely 19, 20. Notably, the first generation of inhibitors exhibited considerable nonspecificity and also targeted other kinases of the AGC family [121]. Whether this is responsible for the adverse effects leading to AMT is unclear. Nevertheless, it encouraged the development of second-generation ROCK and/or MRCK inhibitors such as RKI-18, BDP5290 or DJ4, which show substantially better specificity. Although these inhibitors are widely used in experimental conditions, no *in vivo* data are yet available for RKI-18 [119], BDP5290 [120] or DJ4 [122]. However, DJ4 was found to inhibit the invasiveness of human breast carcinoma MDA-MB-231 cells [122].

### ROCK/PKA/PKB Inhibitors

The report by Sadok *et al.* represents the first evidence that an ROCK/PKA/PKB multikinase inhibitor impairs both ‘amoeboid-like’ and ‘mesenchymal-like’ modes of cancer cell invasion. The compound CCT129254 reduced the motility of melanoma cells *in vivo* and greatly reduced the ability of these cells to colonize the lungs [112]. CCT129254, which has both antimigratory and antimetastatic properties, is among the candidates most likely to meet the requirements of a novel migrastatic drug. Also, the other compound tested, AT13148, was able to inhibit the invasiveness of melanoma cells *in vitro* and *in vivo*; however, because of toxicity in heavily immunocompromised mice, its effect on metastasis was not analyzed. Nevertheless, AT13148 is, to our knowledge, the only ROCK inhibitor in clinical development for oncological indications (reviewed in [123]), and is currently at Phase 1 clinical trial in patients with advanced solid tumors [124]. Interestingly, it is notable that the AT13148 compound showed adverse cardiovascular effects, including vascular smooth muscle contraction, reduction of blood pressure, and tachycardia, although these effects resolved after repeated dosing.

### Drugs Targeting Ion Transport Proteins

Besides cytoskeletal elements and proteins with direct roles in contractility, ion transport proteins have been proposed to be attractive candidate target proteins for interfering with cell migration and/or invasion (reviewed in [125]), since they are easily accessible as membrane proteins and are often overexpressed or activated in cancer. The role of ion transport proteins in migration and/or invasion is mainly attributed to the involvement in the pH- or Ca-dependent regulation of actin cytoskeleton or cell adhesion. Importantly, several clinically widely used drugs are available. However, their anticipated efficacy as antimetastatic drugs has now only begun to be evaluated [125].

## Key Challenge of Migrastatics: Toxicity

As with currently approved anticancer drugs, migrastatic agents that reach the clinical setting could be limited by drug toxicity [126]. Thus, phalloidin and pentabromopseudilin are not discussed in this review. The requirements for low toxicity of migrastatics will be more prominent than with cytostatic drugs, since, to prevent cancer cell invasion, the administration of migrastatics is anticipated to be continuous. However, detailed analysis of various plans of therapy may reveal that intermittent application is possible [127].

The approach of targeting the actin cytoskeleton has been thought for a long time to be too toxic for clinical application [128]. It is clear that targeting actin cytoskeleton dynamics and/or contractility affects many processes in both cancer and normal cells, such as cell migration, division, and exocytosis. In addition, synaptic plasticity relies on F-actin and may be affected by migrastatics [129] as well as by endothelial integrity [130]. Importantly, cell migration is a fundamental step in embryonic development and wound repair [131]. Accordingly, migrastatics may be inappropriate in women with child-bearing potential, and caution should be exercised in patients with diabetes.

There is justified concern that migrastatics will affect immune processes by interfering with both mesenchymal and amoeboid migration of leukocytes. In addition, they can inhibit granule exocytosis-dependent target cell killing by cytotoxic T lymphocytes, as shown for latrunculin A and jasplakinolide [132]. However, in the context of the immune tumor microenvironment, migrastatics could elicit positive therapeutic effects. Recently, it was shown that chondramide A may contribute to an antitumoral microenvironment by depletion of M2 and activation of M1 macrophages. Similarly, ROCK2 inhibition suppresses the M2 phenotype [133]. This suggests that migrastatics could target tumor-associated macrophages in addition to neoplastic cells. Additionally, inhibition of ROCK leads to Fas-ligand overexpression in melanoma cells, resulting in infiltration of leukocytes and reduced tumor growth *in vivo* [134].

Here, we have reviewed examples that demonstrate the feasibility of targeting actin with migrastatics in *in vivo* animal models of tumor metastasis. Although inhibition of cancer cell motility, possibly selective, is a crucial chemotherapeutic target for migrastatic agents, one should be aware of the many physiological functions that are inherently dependent on such capabilities. For this reason, the lowest effective dose must be identified. Moreover, natural products targeting the cytoskeleton as well as synthetic drugs deemed too potent to elicit therapeutic benefit can now be conjugated to an appropriate protein delivery system, thereby limiting the delivery of specific and effective treatments to neoplastic tissue [36].

## Concluding Remarks

We direct recognition to a new class of drugs: the migrastatics. To date, the most promising agents are multikinase inhibitors targeting either ROCK/MRCK or ROCK/PKA/PKB kinases of the AGC family. These inhibitors target a pattern of signaling leading to enhanced cell contractility that is required for all modes of cancer cell invasion. Actin- and contractility-targeting drugs are an intriguing area of pharmacological research, and could revolutionize cancer treatment (see Outstanding Questions). Such drugs have already demonstrated desired effects in many *in vitro* and *in vivo* preclinical studies, and represent promising candidates for clinical evaluation.

It should be emphasized that the goal here is not to replace antiproliferative therapy, but rather complement it. In fact, synergy of migrastatics with antiproliferative cancer drugs appears to be a promising approach for treatment of metastasis (Box 1). Moreover, from recent results, it appears that migrastatics targeting ROCK kinases could themselves have antiproliferative characteristics. It was shown that inhibition of both ROCK isoforms caused severe proliferation defects and loss of both ROCK1 and ROCK2 blocked tumor formation in mice [135].

Box 1 **Synergy of Migrastatics with Other Groups of Anticancer Drugs**

The interactions of migrastatics with other groups of anticancer drugs may offer chances for the synergistic impairment of tumor cells. Migrastatics targeting actin polymerization or dynamics could be used to enhance the efficacy of physicochemical therapeutic approaches resulting in cytoskeletal perturbations, such as X-radiation or sonodynamic therapy 136, 137. The combination of migrastatics with other cytoskeleton-targeting agents could also result in effective chemotherapeutic protocols. Since many migrastatics target cytokinesis, their combination with microtubule-directing agents interfering with mitosis could result in the more efficient inhibition of tumor cell proliferation, as shown *in vitro* for the combination of cytochalasin B and vincristine [138]. Cells exposed to cytochalasin B and also other actin dynamic-targeting migrastatics exhibit significantly increased mitochondrial activity [136], rendering them potentially more vulnerable to mitochondrial metabolism-directed agents. Since tumor cells exposed to migrastatics inhibiting cytokinesis have a highly perturbed cytoskeleton due to the disruption of actin polymerization and multiple nuclei because of high proliferation rates 51, 139, 140, they could be more sensitive to DNA-directed agents, such as alkylators or nucleoside analogs [36]. Intriguingly, migrastatics targeting ROCK kinases could themselves have antiproliferative characteristics. It was shown that inhibition of both ROCK isoforms causes severe proliferation defects and loss of both ROCKI and ROCKII blocked tumor formation in mice [135]. Thus, based on this evidence, we are convinced that migrastatics could complement the current clinical armory, providing more comprehensive and, therefore, more effective therapeutic protocols.

Alt-text: Box 1

Critical assessment of these novel antimetastatic agents is warranted and hopefully will establish new and improved options for the treatment of solid cancer that is consistent with interruption of the natural course of the disease. It is expected that oncology regulations will soon consider guidelines for the development of antimetastatic drugs directed at prevention and treatment [4]. All elements are in place for the entry of migrastatics onto the next stage of anticancer research and development.

Outstanding Questions In solid cancer, does ongoing metastatic activity negate the ‘benefit’ of tumor shrinkage? Why are regulatory end-points of preclinical drug selection still primarily based on tumor shrinkage and not on their antimetastatic activities?

Can recent progress in delivering agents specifically to neoplastic tissues decrease the risk of adverse effects?

Can progress in advanced imaging offer the possibility of tracking stepwise events in the metastatic cascade and could this validate the use of migrastatics?
